# Quasi-In-Situ Analysis of Electrode Top Atomic Layers via High-Sensitivity Low-Energy Ion Scattering and Potential-Controlled Sample Transfer

**DOI:** 10.1021/acs.chemmater.5c02629

**Published:** 2026-05-13

**Authors:** Haoran Ding, Nathanael C. Ramos, Anish Parulekar, Adam Holewinski

**Affiliations:** † Department of Chemical and Biological Engineering, 1877 University of Colorado, Boulder, Colorado 80309, United States; ‡ Renewable and Sustainable Energy Institute, University of Colorado, Boulder, Colorado 80309, United States; § Department of Physics, University of Colorado, Boulder, Colorado 80309, United States

## Abstract

Electrocatalytic reactions involve interfacial interactions between the surfaces of electrodes and reactive species at an electrolyte interface. There are presently no universal or unambiguous methods to directly assay the active top atomic layer composition that influences the reactivity of these electrodes under relevant operating conditions. Low-energy ion scattering (LEIS) spectroscopy is a surface characterization technique that yields compositional analysis of the outermost atomic layer of a material, but it must be performed in ultrahigh vacuum (UHV). Application of LEIS measurements to electrochemical materials that are removed from ambient liquid-phase environments thus leaves an open question as to whether the surface that is transferred to UHV is truly the surface that manifested during the electrochemical reaction. Toward the goal of preserving the active surface state, we developed a sample transfer workflow for LEIS enabling air-free removal and drying of an electrode from an electrochemical cell while maintaining control of the potential using an auxiliary electrode. The potential-controlled emersion method was demonstrated to give distinct potential-dependent surface compositions for a Cu–Pd alloy relative to removal after uncontrolled return to open-circuit potential. A Cu-enriched surface was found at anodic potential and a Pd-enriched surface at cathodic potential, suggesting that the approach can be used to retain representative atomic configurations during transfer. Since adsorbates will often persist from the reaction environment, conventional sample pretreatment methods for removal, including atomic O and atomic H exposure, were also contrasted. Both methods were found to differ with results from incidental low-dose depth profiling by the LEIS primary ion source, which removes adventitious species and surface atoms during the course of repeated measurements. These depth profiles were found to be sensitive to sample history and thus qualitatively informative, despite the possible changes induced by ion damage. The results exhibit (i) the need for complete control over the polarization state of the sample at all times (no excursions to open circuit during transfer) and (ii) the utility of low-dose depth profiling to capture changes in the near-surface composition.

## Graphical abstract

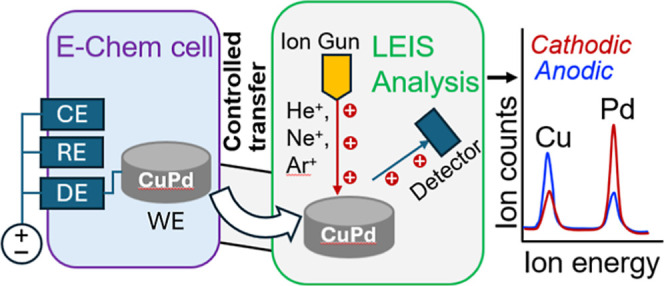

## Introduction

The study of surface composition is of great significance in various science and engineering fields such as heterogeneous (electro)­catalysis,^1,2^ batteries,^3,4^ atomic layer deposition^5^ and coatings,^6^ and semiconductor fabrication.^7^ Low-energy ion scattering (LEIS) is a surface characterization technique involving the bombardment of a surface with noble gas ions (usually He^+^, Ne^+^, or Ar^+^), also known as primary ions, which backscatter from the sample.^8–12^ Typically, primary ion energies range from 1 to 8 keV, which is much lower than other ion scattering techniques such as Rutherford backscattering.^13^ LEIS is thus largely sensitive to the outermost atomic layer of a material, making it extremely surface-enhanced compared with other “surface sensitive” characterization methods like X-ray photoelectron spectroscopy (XPS), which probes a depth of several nanometers.^14^ The interaction between the primary ions and surface atoms can be roughly considered as an elastic collision, meaning that the chemical environment of the target atom has minimal influence on the sensitivity factor for the element (avoiding matrix effects whereby the presence of one component affects the quantification of other components).^9^ The detector measures the velocity (energy) of the backscattered primary ions, and the heavier the surface atom, the higher the energy of the backscattered primary ions.^8^ The optimal primary ion choice depends on the sample: surface atoms have to be heavier than the primary ions or no backscattering can occur; at the same time, if the mass difference is too large then the primary ion will not transfer sufficient momentum to yield well-resolved signals between surface atoms of similar masses.^12^ By plotting the counts of backscattered primary ions versus their energy, the LEIS spectrum is obtained (Figure 1a). By also utilizing a modern detector with high collection efficiency, so-called high-sensitivity LEIS (HS-LEIS) can be performed with doses below 1% of a monolayer, making the measurement relatively nondestructive.^8^

**Figure 1:**
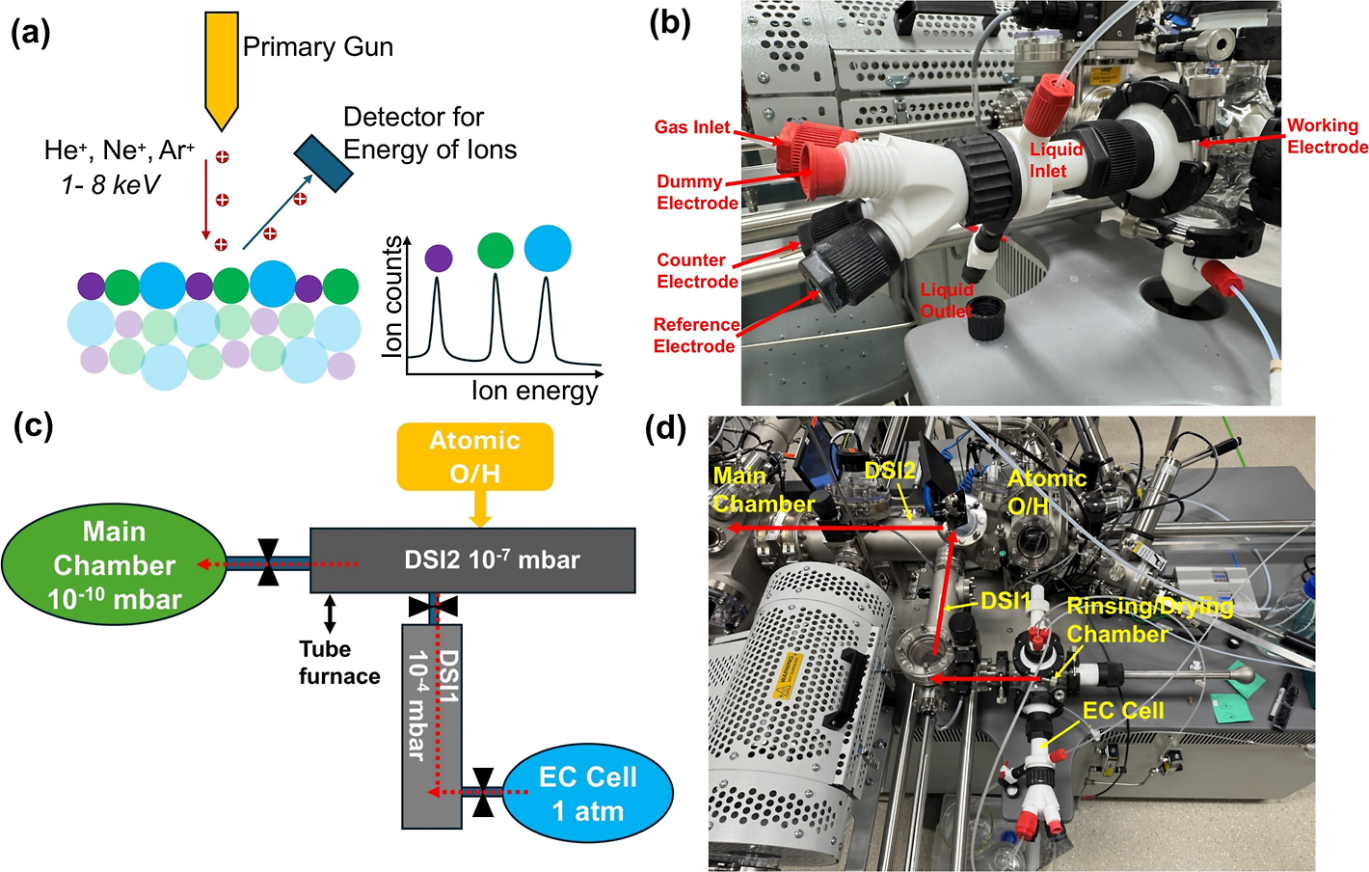
(a) Basic principles of the LEIS technique. (b) Photo of electrochemical cell. (c) Schematic of experimental setup with cell coupled to LEIS vacuum system. Red dashed arrows show the sample loading path. (d) Photo of experimental setup. Red solid arrows show the sample loading path.

Electrochemistry studies charge transfer at the interface between an electrode and an electrolyte. Electro*catalytic* applications (e.g., green hydrogen production,^15^ fuel cells,^16^ CO_2_ reduction,^17^ and other electrosynthesis reactions^18,19^ ) are particularly sensitive to the top atomic layer composition of the electrode, as reactant species make and break bonds with the surface during these reactions. Many previous works have successfully applied surface characterization techniques such as XPS,^22^ Auger electron spectroscopy,^23^ and attenuated total reflectance–surface-enhanced infrared absorption spectroscopy (ATR-SEIRAS)^20,21^ to electrochemical materials. However, these techniques are not as surface sensitive as LEIS.^14^ As a notable point of comparison, trace adventitious contaminants on a material’s surface may only show up as a small C 1s peak in XPS, but these same species can significantly undermine the acquisition of interpretable LEIS spectra. Therefore, additional care needs to be taken to preserve or clean the surface before a LEIS measurement. Furthermore, any pretreatment method needs to be carefully evaluated because it may also change the surface composition, generating an unrepresentative spectrum.

In this study, we coupled an electrochemical cell at ambient pressure (Figure 1b) with HS-LEIS through a vacuum sample transfer path (chambers labeled DSI1 and DSI2, where DSI refers to “dual stage intro”, Figure 1c,d) to avoid exposure to air.^24–26^ Sputter-cleaned samples of pure Cu and a CuPd alloy were used as model electrodes to explore the effects of electrochemical polarization on signal recoverability (adsorbate retention) as well as top atomic layer segregation. Benchmark studies were first performed to determine sensitivity factors and assess the influence of common cleaning methods such as atomic O and atomic H on the measurement. These were found to alter the surface composition relative to sputter-cleaned samples and thus not be appropriate for cleaning due to the reactivity of the surfaces. Low-dose ion milling during the course of repeated measurements using Ne^+^ from the primary ion gun was found to provide a qualitative depth profile through the first few atomic layers that is sensitive to the sample history. It was found that the surface and near-surface profile of a CuPd alloy differs under anodic versus cathodic polarization and that these differences can only be preserved with the use of an auxiliary “dummy” electrode to maintain the potential while removing and drying the sample surface. The results exhibit (i) the need for complete control over the polarization state of the sample at all times (no excursions to open-circuit potential during transfer) and (ii) the qualitative utility of low-dose depth profile measurements using the HS-LEIS primary ion source.

## Experimental Methods

All LEIS measurements were performed with an IONTOF Qtac 100 HS-LEIS instrument. A photo of the instrumental setup is shown in Figure 1d. The sample loading path consists of three sections: the DSI1 chamber with ∼10^–4^ mbar vacuum; the DSI2 chamber with ∼10^–7^ mbar vacuum; and the main analysis chamber with ultrahigh vacuum (∼10^–10^ mbar), where measurements take place. LEIS measurements were performed with a primary gun emitting He^+^ or Ne^+^. The primary ion energy (normal to sample stage) was set to 3 keV for He^+^ and 5 keV for Ne^+^ with the dose per measurement typically set to 5 × 10^13^ ion/cm^2^ for He^+^ and 1 × 10^13^ ion/cm^2^ for Ne^+^ (changes to specific conditions will be noted where appropriate below). A secondary sputter gun was configured for a higher dose rate (lower limit ∼10^15^ ion/cm^2^ in 10 s) of 2 keV Ar^+^ at 60° incidence; this sputtering condition was used to rapidly remove multiple surface layers to obtain a clean and reproducible surface for benchmarking. The Qtac 100 is equipped with a double toroidal analyzer to measure the backscattered ion energy.^27,28^ A built-in atomic O/H source (IONTOF) was also used where noted. IONTOF SurfaceLab software was used in peak identification and integration.

LEIS quantifications of Cu and Pd were achieved by calibration using a pure Cu electrode disk (99.99%, Pine Research) as well as a Pd disk (99.99%, Goodfellow). The sensitivity factors were determined as the LEIS response (integrated peak area) per unit ion dose on the pure sputter-cleaned surface of the single element. Normalized theoretical yields were then reported for all samples using the formula:

$$normalized\;theoretical\;yield\;of\;X=\frac{measured\;response\;\left(counts/dose\right)}{sensitivity\;factor\;of\;X}$$

Elemental ratios, where shown, were computed as the ratio of the normalized theoretical yields. Due to some drift in the primary beam ion current over long measurements, the incident current was treated as an average of the initial and final current. While this creates some uncertainty in absolute quantifications during depth profiling, elemental ratios are not sensitive to this effect.

The electrochemical cell was from SPECS Surface Nano Analysis GmbH with a custom interface connecting to the vacuum transfer path through the DSI1 chamber and a glass intermediate chamber, where rinsing and drying of the working electrode (WE) are performed (Figure 1b). The cell has an inlet and outlet for liquid dosing as well as a WE interface and three additional electrode ports for a counter electrode, a reference electrode, and a fourth electrode or other probe. A sample loading rod is equipped to position the WE and interface with another transfer rod to reach the DSI1 chamber. A polycrystalline Cu disk (99.99%, same as used for calibration) and a 40% Cu–60% Pd alloy foil (10 mm × 10 mm, Goodfellow) were each used as working electrodes. Each WE was embedded into a PTFE electrode housing with a feedthrough at the back that allowed electrical contact with the metal LEIS sample holder via a gold pin. The WE is positioned such that the PTFE housing can be pressed against an O-ring to seal the electrochemical cell. A CAD drawing with additional detail is shown in Figure S1.

All electrochemical tests were performed in aqueous electrolyte solutions made from 18 MΩ water and 0.5 M NaHCO_3_ (Sigma-Aldrich, >99.5%, solution pH = 8), continuously purged with Ar (Airgas, 99.9999%). Liquid pumping to the cell was controlled by a Metrohm Eco Dosimat dosing unit. A graphite rod was used as the counter electrode. An Ag/AgCl (sat. KCl) was used as the reference electrode. A Cu wire was used as the dummy electrode (DE). All electrochemical controls and measurements were performed with a CHI842B potentiostat (CH Instruments). The HS-LEIS instrument also had connections with a tube furnace and a transfer chamber to interface with a Kratos Axis Supra XPS, though not used in this study.

## Results and Discussion

### LEIS of Monometallic Cu Surface

A pure Cu disk electrode was first analyzed by LEIS to evaluate the effect of pretreatments on the state of the surface and signal recoverability. Beginning with introduction of the sample from the ambient atmosphere without any pretreatment (sample “Pre”), LEIS spectra using both 3 keV He^+^ and 5 keV Ne^+^ show negligible response (Figure 2a–c). This is due to (i) oxidation of the Cu and (ii) blockage of the Cu-oxide by other contaminants that adsorb to the surface during ambient air exposure (trace organics). These effects are illustrated further in depth-profiles alternating 3 keV He^+^ LEIS measurements with 2 keV Ar^+^ sputtering cycles on Cu under different exposure conditions in Figures S2 and S3. We note that even with the light He^+^ primary ions, hydrocarbon-based contaminants are not resolved as peaks; these manifest mainly through a baseline increase at very low energy from emitted secondary ions of light elements (mostly hydrogen). After deep sputter cleaning with 2 keV Ar^+^, the maximum signal intensity for “clean” Cu is observed (Figure S4) and establishes the sensitivity factor (60,574 cts/nC with 5 keV Ne^+^).

**Figure 2:**
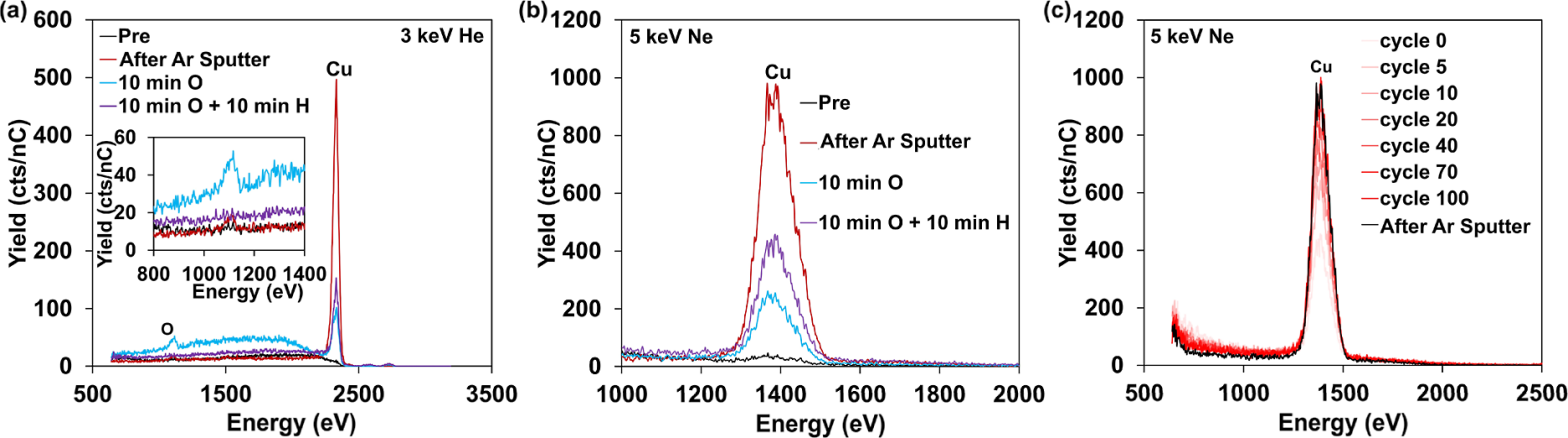
LEIS of pure Cu using (a) 3 keV He^+^ and (b) 5 keV Ne^+^ in sequential analyses: as-introduced (black), after sputter cleaning (red), after sputter clean + 10 min atomic O (blue), and after fresh sputter clean + 10 min atomic O + 10 min atomic H (purple); inset of (a) shows oxygen peak region more closely; and (c) LEIS spectra through 100 cycles of 10^13^ ion/cm^2^ 5 keV Ne^+^ depth profiling for slow milling of Cu surface layers after final treatment of (a)/(b).

Next, the clean Cu disk was transferred to the DSI2 chamber, where the impact of atomic O and H pretreatments was evaluated. These treatments, and particularly atomic O, have been used effectively for cleaning noble metals and metal oxides introduced with contaminants. However, these exposures can alter the surface of more reactive metals. After 10 min under the atomic O source, LEIS of the Cu sample (reintroduced to the analysis chamber) shows that a surface oxide layer is formed over a significant fraction of Cu atoms, resulting in a smaller Cu peak and the emergence of an O peak in the 3 keV He^+^ spectrum. A visible shoulder in intensity to the left of the Cu peak in the He^+^ spectrum after O-exposure comes from a reionization background, which is enhanced (compared to metallic Cu) due to strong charge transfer between He^+^ and O. The He^+^ is neutralized, scatters off subsurface Cu, then reionizes and is detected with lower energy. This illustrates one of the known instances of a matrix effect in LEIS (O affecting the sensitivity of He^+^ to metals).^9,29–31^ The small peak at slightly higher energy (2600 to 2700 eV) is assigned to double or multiple scattering phenomena.^8^

Further pretreatment by 10 min under the atomic H source reduced the oxide and exposed more Cu, but the Cu peak did not return to the full intensity of the sputter-cleaned surface (Figure 2a,b). To gain better insight into the dynamics between O and H treatments, we also performed a longer 30 min atomic O exposure, followed by 10, 20, and 30 min of atomic H treatment (Figure S5). Differences in the resultant spectra after each of these treatments were marginal, suggesting that much of the adsorbed O is too strongly bound for effective removal by H. Some H may also remain adsorbed and attenuate signal. Given the strong reactivity associated with atomic O and the inability to remove by atomic H, it was determined that this approach is not appropriate for cleaning Cu, as may be expected from its chemical reactivity.

Since atomic O/H treatment cannot be used for cleaning metallic Cu, the evolution of the spectrum under mild surface milling during analysis was explored as a means to probe the extent to which the surface layer(s) become modified. In contrast to sputtering by the dedicated Ar^+^ sputter gun, the primary probe ion gun can deliver extremely small ion doses. Estimating 10^15^ atoms/cm^2^ for a solid metal surface and estimating a sputter yield of 1 for 5 keV Ne^+^ (removing 1 surface atom by 1 bombarding ion),^32^ a dose of 10^13^ ion/cm^2^ equates roughly to the removal of around 1% of a full monolayer. Thus, through a sequence of several hundred low-dose cycles with spectrum acquisition at each cycle, an assay of the top few atomic layers can be acquired with qualitative depth resolution. After the atomic O and atomic H pretreatments, the Cu surface was analyzed through 100 cycles of 10^13^ Ne^+^ ion/cm^2^ milling (Figure 2c). The O signal decreases, while the Cu signal increases, and after 100 cycles, the Cu peak area was close to the clean Cu surface seen after high-dose Ar^+^ sputtering (black trace). Similar effects of atomic O/H and Ne^+^ milling were also observed using a pure Pd foil (Figure S6).

### LEIS of CuPd Alloy

A Cu_40_Pd_60_ bulk alloy was next used to investigate differences in surface composition resulting from varying pretreatments as well as exposure to electrochemical reaction conditions. The composition was chosen because the monometallic sputter yields of Cu and Pd are relatively similar,^33^ although previous work has suggested this alloy will somewhat favor loss of Cu under ion bombardment due to a weaker binding energy at the alloy surface.^34–36^ While preferential sputtering may thus play a role in observations, it should not influence strong qualitative trends at low damage levels.

Using the sensitivity factors from pure Cu and pure Pd surfaces (Cu: 60,574 cts/nC; Pd: 62,752 cts/nC, both with 5 keV Ne^+^), the Ar^+^-sputtered CuPd alloy surface gave a LEIS surface composition of Cu_47_Pd_53_ (Figure 3a). This is near to the nominal composition, but considering that Cu is more likely to become depleted by sputtering, suggests that Pd may preferentially recontaminate at a higher rate during the switch between ion sources (1–2 min) and subsequent spectrum acquisition. Further low-dose Ne^+^ milling (dosing ∼10^13^ ion/cm^2^ per spectrum, two spectra per minute) (Figure S7) reinforces this interpretation: relative to the Ar^+^ sputter-cleaned starting point, the total (all element) signal intensity decreases toward a new stable value over about 50 cycles (5 × 10^14^ ion/cm^2^). This suggests potential effects—due to the protracted timespan of Ne^+^ milling (several hours)—from recontamination of residual vacuum species, readsorption of sputtered species and/or surface diffusion from outside the sputtered area. The total signal decrease is manifested through a drop in Pd signal while Cu holds more constant, leading to a bulk LEIS Cu/Pd ratio of 1.2. Ne^+^ milling of pure Cu and pure Pd also shows a faster signal decay rate (after initial sputter clean) for Pd relative to Cu, suggesting preferential contamination on Pd (Figure S7c). Common residual gas composition in UHV includes hydrogen (diffusing out from the steel chamber walls) and carbon species (mainly CO, CH_4_, and CO_2_) that can form and desorb from ion gauges as residual gases are collected.^37^ Preferential contamination may explain the elevated steady state Cu/Pd ratio seen on the alloy during Ne^+^ milling. Despite any complexity related to preferential sputter and preferential contamination, it is worth again emphasizing that changes to depth profiles across sample treatments can qualitatively inform on chemical effects of interest.

**Figure 3:**
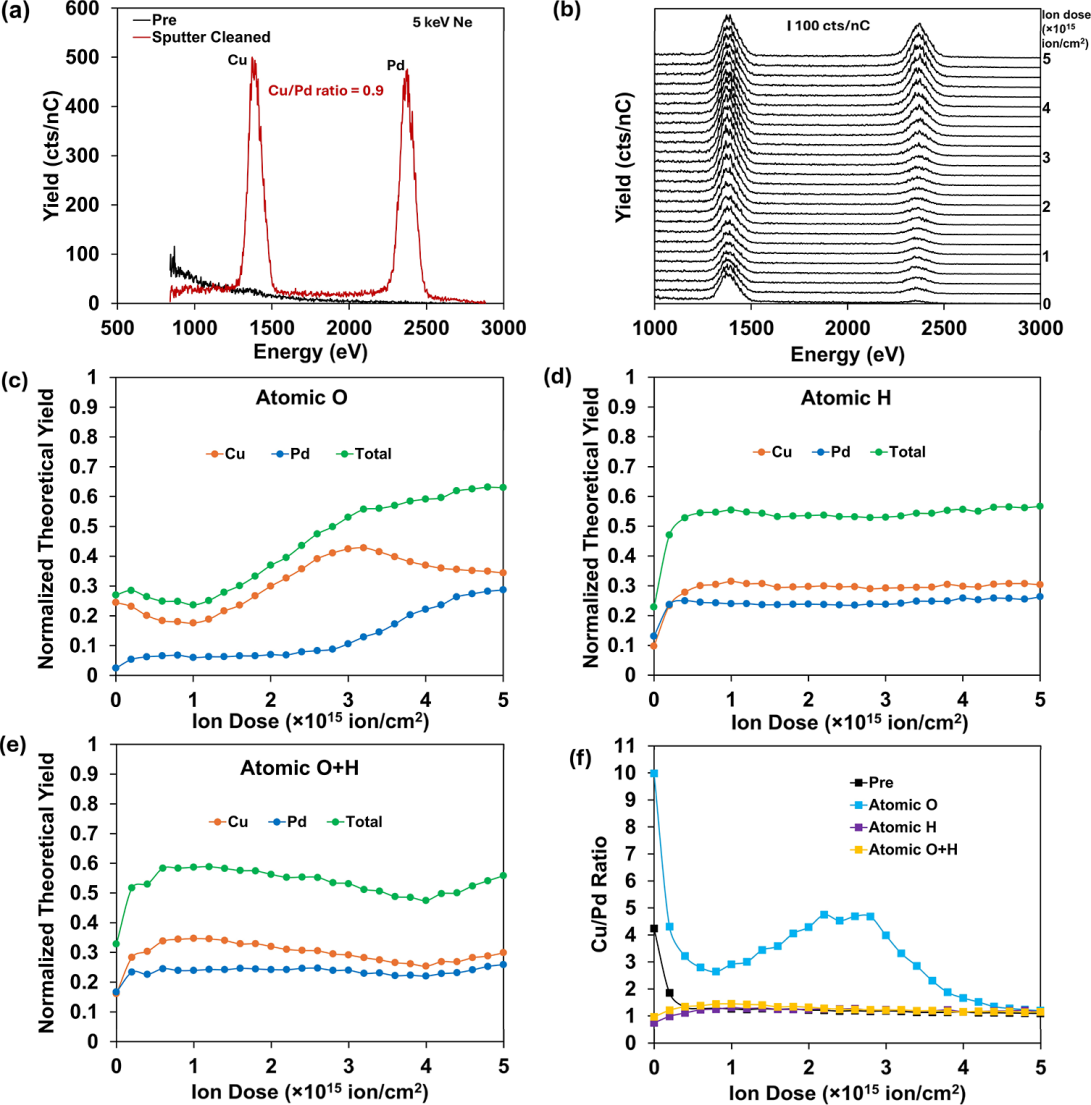
(a) LEIS of CuPd alloy with no pretreatment and after Ar^+^ sputter cleaning; (b) LEIS of CuPd alloy through 500 cycles of Ne^+^ depth profile (10^13^ ion/cm^2^/cycle), taken after 10 min atomic O pretreatment of the sputter-cleaned surface; (c–e) LEIS quantification of CuPd foil through 500 cycles of Ne^+^ after (c) 10 min atomic O; (d) 10 min atomic H; and (e) 10 min atomic O + 10 min atomic H. Normalized theoretical yield (*y*-axis) refers to the signal intensity compared to the maximum signal determined by sensitivity factors of the sputter-cleaned pure metals. Ne^+^ dose per cycle: 10^13^ ion/cm^2^. (f) Comparison of Cu/Pd ratio changes during Ne^+^ depth profiles from (c–e).

Chemically induced surface segregation was next explored through treating the sputter-cleaned alloy with atomic O and atomic H. Figure 3b shows a Ne^+^ depth profile experiment on the CuPd alloy after 10 min atomic O pretreatment. Asynchronous increases in both Cu and Pd signals can be seen, and the apparent surface concentrations of Cu and Pd (normalized to the sensitivity factors from sputter-cleaned pure metals) are presented as a function of ion dose in Figure 3c (each cycle has an ion dose of 10^13^ ion/cm^2^). It is worth noting that the steady state total normalized theoretical yield always stays below 1, suggesting effects from recontamination of residual vacuum species, readsorption of sputtered species, and surface diffusion from outside the sputtered area. The Cu/Pd ratio (compiled in Figure 3f) initially increases to 10.0 from the atomic O exposure. Up to ∼100 measurement cycles (10^15^ ion/cm^2^ cumulative dose), there is a decrease in Cu signal, though it still remains larger than the Pd signal. The Cu signal then increases again from cycles 100 to 300 before a sharp increase in Pd pushes the ratio toward its bulk value. The reasons for the dip in Cu signal over the first 100 cycles are unclear, though might relate to the specific Cu-oxide surface structure and/or vacancy creation during the reaction with atomic O. Artifacts such as drift in beam current or readsorbing residual species are not expected to contribute as the total signal intensity remains constant while the Cu/Pd ratio changes. At greater depths, the oxidation penetration decreases and may lead to a stoichiometry shift, such as from CuO to Cu_2_O (although such phase domain assignments are not meaningful for such a shallow surface assay).^38^ It is worth noting in Figure 3f that the surface of the “Pre” sample (no pretreatment) also shows Cu enrichment, likely caused by the contact with molecular O_2_ in the air (full profile in Figure S8). Atomic H pretreatment, as well as pretreatment first by atomic O followed by atomic H, both result in Pd enrichment at the outermost layer (Figure 3d–f). The Pd enrichment could be explained by the higher affinity of Pd for H. In all cases, the Cu/Pd ratio approaches the bulk value after about 400 cycles.

### Ex Situ LEIS of CuPd Alloy after Electrochemical Polarization

The effects of electrochemical polarization on the surface of the CuPd alloy in aqueous electrolyte were next tested. It was hypothesized that, as seen above, oxidative environments (anodic polarization) would favor segregation of Cu to the top atomic layers, while reducing environments (cathodic polarization) would favor segregation of Pd. Oxidation can also lead to dissolution of metals—here Cu in particular—and in aqueous solutions, this effect will be pH-dependent. Neutral to moderately alkaline pH minimizes the solubility (though still nonzero, Figure S9a), so all experiments were carried out in 0.5 M NaHCO_3_ (pH 8). Sufficient rinsing of the electrode surface with DI water (we used ∼150 mL) is necessary in removing Na^+^ contamination; otherwise, the salt will precipitate on the surface and reduce metal signals (Figure S10).

Cyclic voltammetry shown in Figure 4a shows that oxidation of the CuPd electrode is milder and pushed to a higher potential than for the pure Cu electrode; in contrast, the alloy exhibits significant HER activity approaching −1 V, while monometallic Cu is inactive at these potentials. The two cathodic peaks at −0.2 V and −0.6 V on Cu are attributed to the two-step reduction of Cu^2+^ to Cu.^39^ On the CuPd alloy, the anodic peak at −0.4 V is attributed to the hydrogen oxidation reaction, while the cathodic peak at 0 V is attributed to the reduction of Cu species. On the basis of these characteristics, fixed potentials of −1, 0, and 0.5 V were chosen to compare the surface structure by LEIS.

**Figure 4:**
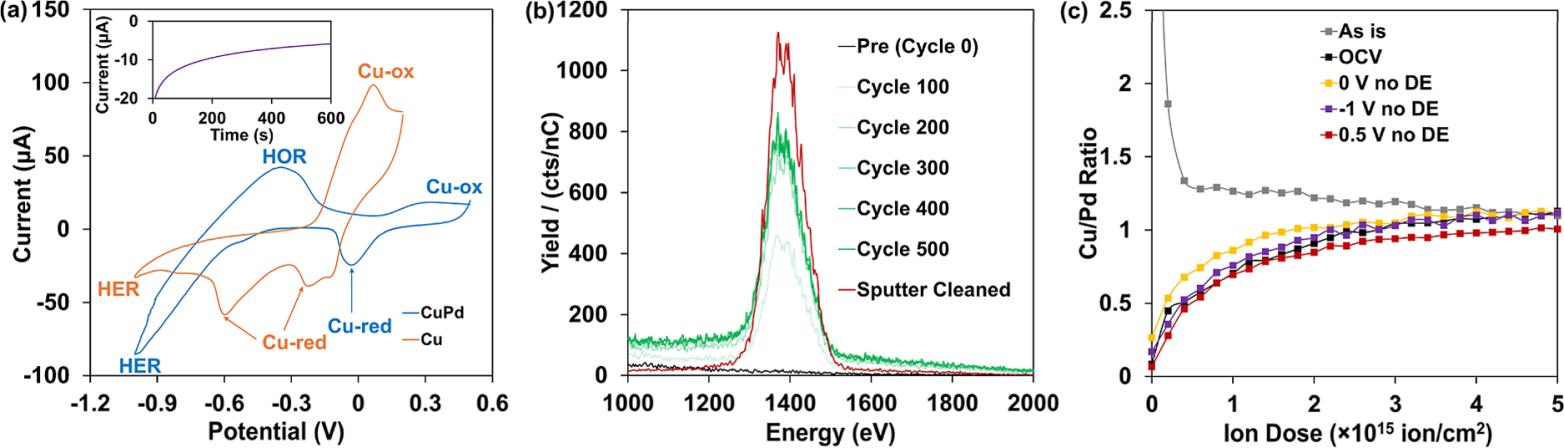
(a) CV (20 mV/s) of Cu and CuPd alloy electrode in 0.5 M NaHCO_3_ solution; the inset shows a 10 min potentiostatic *I*–*t* curve at −1 V for the CuPd alloy electrode. Potentials are relative to Ag/AgCl reference electrode. (b) Series of 5 keV Ne^+^ LEIS milling cycle spectra (10^13^ ion/cm^2^/cycle) of Cu electrode after −1.0 V hold for 10 min and transfer without potential control; (c) Cu/Pd ratio as a function of Ne^+^ milling cycles and different potentials on a CuPd electrode without potential-controlled transfer conditions.

LEIS spectra were acquired for samples polarized using a 3-electrode cell coupled to the vacuum system (cf. Figure 1 and Methods). Figure 4b shows 5 keV Ne^+^ LEIS of the pure Cu electrode after holding at −1.0 V potential for 10 min. After the electrochemical polarization and sample transfer (with no potential control during removal), the Cu signal was almost completely suppressed. Aside from surface oxidation from reaction with water after removing potential control, contamination could come from organic impurities in the electrolyte, impurities in the Ar purge gas, and/or organic vapors in the DSI vacuum zones. We also note that surface roughness, while capable of mild signal attenuation relative to single crystal references,^8^ did not change significantly according to double-layer capacitance measurements before and after electrochemical experiments (Figure S11). By using Ne^+^ milling, after 3 × 10^15^ ion/cm^2^ cumulative dose, the Cu signal rose and reached a steady state with only 0.8% change from 3 × 10^15^ ion/cm^2^ dose to 4 × 10^15^ dose (Figure S12).

Electrochemical studies were next performed with the CuPd alloy electrode. In the absence of any control over potential during sample transfer, all polarization conditions resulted in a similar distribution of elements at the measured surface. Figures S13 and 4c show LEIS profiles after potential holds at −1 V, 0 V, and 0.5 V, as well as open circuit potential (OCP), which was found to sit roughly around 0.1 V. In each case, post-potential-control exposures and/or sample transfer led to a surface with suppressed metal peaks, as described above for pure Cu. Ne^+^ depth profiling was then used to help to obtain information from beneath the contaminants. The apparent metal surface Cu/Pd ratio after each polarization condition gave a lower Cu/Pd ratio than the bulk (Figure 4c), in contrast to the as-received CuPd alloy (Figure 3f). After several hundred milling cycles (each 10^13^ ion/cm^2^), the Cu/Pd ratio returned to the bulk ratio in all cases.

While the loss of Cu signal at the surface described above may initially be counterintuitive, in particular for exposure to cathodic conditions that should not allow oxidation or leaching, it must be recognized that the sample will polarize to OCP during transfer if the electrode is simply disconnected while in contact with the solution. Thus, any loss of potential control may lead to electrochemical behaviors that are not representative of the original condition. Leaching and/or preferential adsorption of differing species (hydroxide, electrolyte anions, etc.) could thus still play a role in the observed elemental ratios, even when initially polarizing to a strong cathodic potential.

### Quasi-In-Situ LEIS of Electrodes with Potential-Controlled Transfer

To minimize the effect of transitions to OCP, a secondary WE (a dummy electrode) was connected to the WE lead to maintain potential control when the WE was emersed from the electrolyte. A Cu wire was used as the DE. To ensure there are no transient voltage excursions that influence the surface, the DE must always be connected to the WE, from the moment the WE touches the electrolyte until the WE surface is fully dried under the Ar stream. After drying, the surface is assumed to be static, as the atmosphere is pumped from Ar to UHV. The DE experimental configuration and workflow are illustrated in Figure 5a. The current vs time (*I*–*t*) curve during the first 20 min of a representative DE experiment of CuPd alloy at −1 V is shown Figure 5b, along with labels at the time of critical events.

**Figure 5:**
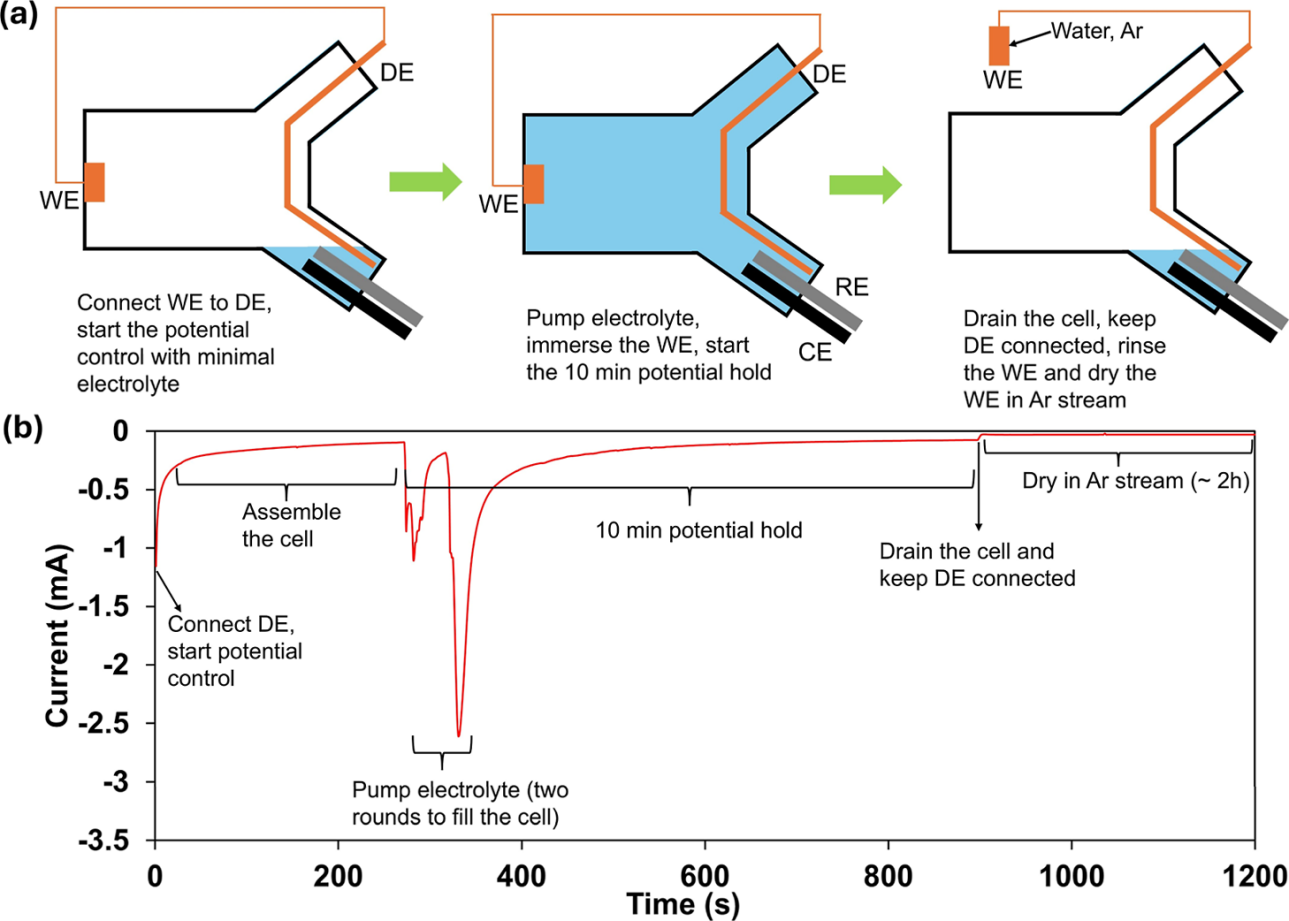
(a) Illustration of setup and steps of DE experiments; (b) *I*–*t* curve of a DE experiment with CuPd alloy at −1 V. Only the first 20 min (of 2 h) is shown as the remainder is continuation of potential-controlled drying in Ar stream.

LEIS results shown in Figure 6 exhibit significant differences between cases with and without DE control. At −1 V (Figure 6a), the Cu/Pd ratio indicates that the surface still appears Pd-enriched, but the profile has a notably sharper transition with ion dose compared to measurements without the DE. This may be interpreted to suggest that there is no longer leaching of Cu, and the subsurface does not become depleted to the same degree. Interaction with adsorbing hydrogen still drives Pd segregation to the surface.

**Figure 6:**
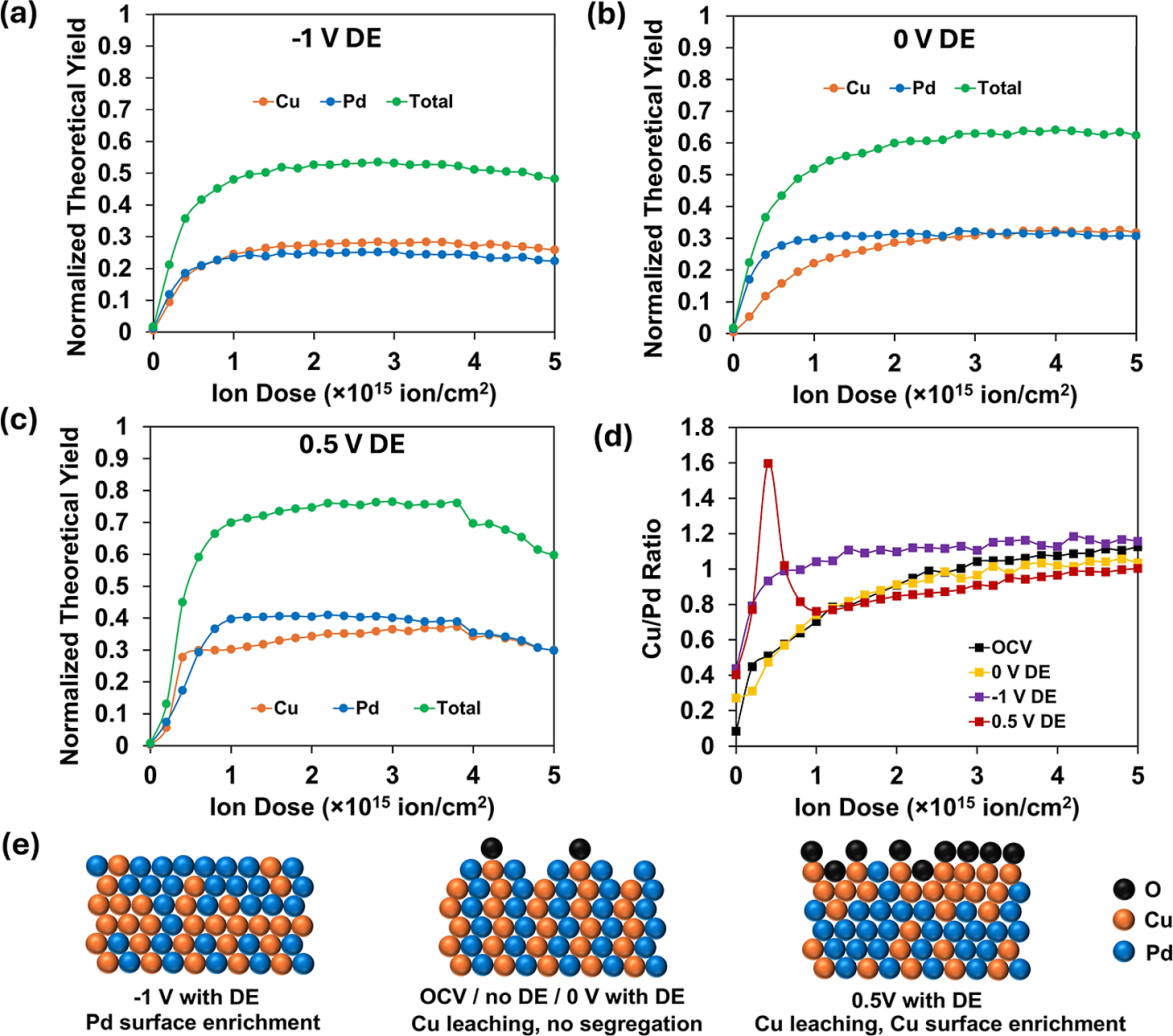
LEIS results after electrochemical polarization of CuPd alloy surface with potential-controlled transfer at (a) −1 V; (b) 0 V; and (c) 0.5 V. Normalized theoretical yield (*y*-axis) refers to the signal intensity compared to the maximum signal determined by sensitivity factors of the sputter-cleaned pure metals. (d) Cu/Pd ratio after electrochemical polarization on CuPd alloy surface at different potentials. Ne^+^ dose per cycle: 10^13^ ion/cm^2^. (e) Hypothesized state of the surface at different potentials derived from the LEIS results.

The experiment at 0.5 V (Figure 6c) with potential control most notably shows a dramatically different composition in the surface region than its non-DE counterpart. Here, after milling through contamination, there is a sharp spike in the Cu/Pd ratio, suggesting that anodic potential does indeed drive Cu to the surface, much like exposure to atmospheric O_2_ or atomic O. At 0 V (near the OCP), differences with the uncontrolled transfer potential are minimal, again suggesting leaching of surface Cu (Figure 6b). Direct overlays of the Cu/Pd profile with and without DE control are shown in Figure S14.

What is perhaps counterintuitive in light of these observations is that the previously shown result, in which a 0.5 V hold was followed by return to OCP (Figure 4c), did not yield Cu enrichment at the surface. To rationalize this, we hypothesize that some differences may exist in the competition between oxidation and dissolution of near-surface Cu at each potential. From the Pourbaix diagram of Cu (Figure S9b), the dominant oxidation pathway at 0 V at pH 8 would be to Cu_2_O; however, the solubility of Cu­(I) species at this pH is still roughly 1.4 nM (Figure S9a), which greatly exceeds the concentration that a few monolayers of material could create in the cell. Leaching is thus expected to contribute, though the kinetics of this process are not established. We hypothesize that at 0.5 V, the driving force for oxidation is sufficient to maintain an oxide layer, which can be replenished faster than it leaches away. At OCP (∼0.1 V), surface domains of Cu can oxidize, but oxygen does not penetrate more deeply or promote the diffusion of subsurface Cu outward. The surface oxide consequently dissolves and forms a Pd or alloy domain-terminated surface that is stable against further oxidation at OCP. Figure 6e schematically illustrates the proposed influence of potential.

## Conclusions

In this study, we successfully combined HS-LEIS with an electrochemical cell to realize quasi-in situ top atomic layer electrode surface analysis. We developed a workflow for the transfer of electrodes to UHV under potential control and demonstrated this approach to uniquely identify the surface composition of a CuPd alloy as a function of the electrochemical polarization. Anodic and cathodic polarization led to Cu-enriched and Pd-enriched surfaces, respectively. It was further found that for reactive samples such as CuPd, conventional sample cleaning methods including atomic O and H exposure do not result in reliable representations of the pre-existing surface. Low-dose Ne^+^ milling was explored as an alternative for removing adventitious species during the LEIS measurement. This contribution shows the need for complete control over the polarization state of the sample at all times (no excursions to OCP) as well as the utility of low-dose depth profiling to analyze top atomic layer surface segregation.

The approach developed in this study may further be applied to a wide variety of electrochemical systems. CuPd alloys are often studied in the context of electrochemical CO_2_ reduction, as are a broad variety of other alloys and multicomponent materials.^40–44^ Understanding the dynamic surface chemistry of such materials is likewise fundamental to designing catalysts for hydrogen evolution, water splitting, and essentially all electrocatalytic processes.^45–48^ Similar questions of dynamic surface chemistry appear in electrochemical energy storage applications such as Li and beyond-Li battery technologies,^45,46^ supercapacitors,^47^ and electrochemical sensors.^48^

## Supplementary Material



## Notes

Additional experimental details; CAD drawings of the electrochemical cell; LEIS depth profiles of electrodes under additional pretreatment and electrochemical conditions; solubility and Pourbaix diagrams for Cu; and electrode surface area characterization (PDF)
