# Supplementary material for: Quasi-In-Situ Analysis of Electrode Top Atomic Layers via High-Sensitivity Low-Energy Ion Scattering and Potential-Controlled Sample Transfer
Source: Chem Mater. 2026 May 13;38(10):4924–32. doi: 10.1021/acs.chemmater.5c02629 (PMC13217553; doi:10.1021/acs.chemmater.5c02629)
Supplement: Supplementary file 1 [file cm5c02629_si_001.pdf]

Supporting Information

Quasi-In-situ Analysis of Electrode Top Atomic Layers via High-Sensitivity Low Energy Ion Scattering and Potential-Controlled Sample Transfer

Haoran Ding[1,2], Nathanael C. Ramos[1,2], Anish Parulekar[1,3] and Adam Holewinski[1,2]*

[1]*Department of Chemical and Biological Engineering, University of Colorado, Boulder, Colorado 80309, United States;* [2]*Renewable and Sustainable Energy Institute, University of Colorado, Boulder, Colorado 80309, United States;* [3]*Department of Physics, University of Colorado, Boulder, Colorado 80309, United States;*

*Corresponding author: adam.holewinski@colorado.edu

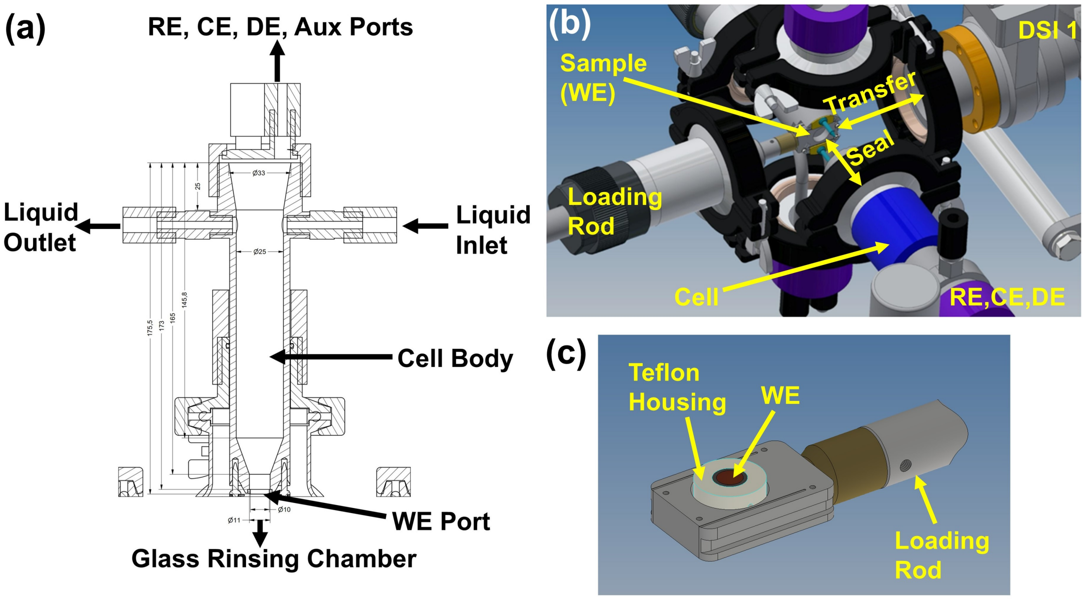

Figure S1. (a) CAD diagram and (b) 3D rendering of the electrochemical cell and (c) electrochemical sample (working electrode) holder.

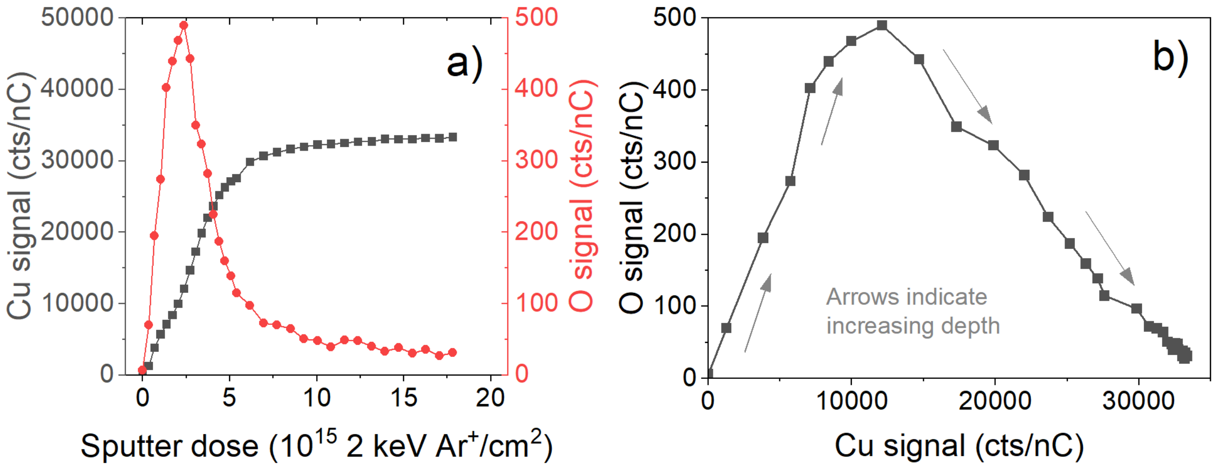

Figure S2. Sputter depth profile of a Cu foil taken from atmosphere. (a) 3 keV He^+ LEIS signal of Cu (left, black) and O (right, red) as a function of 2 keV Ar^+ sputter dose (He^+ spectra acquired alternating with Ar^+ sputter). (b) 3 keV He^+ signal from O (y-axis) plotted against Cu signal (x-axis), where each dot represents a progressive point of the depth profile in (a). Initially, there is no He^+ signal due to atmospheric contamination (adventitious carbon that does not show a peak). With increasing Ar^+ dose, contamination is milled through revealing an O rich surface. Both O and Cu signal increase initially, followed by a peak in O signal that is correlated with the full removal of contamination. Further Ar^+ sputtering reveals a linear relationship between O and Cu suggesting O removal reveals Cu. At high Ar^+ dose, the surface becomes all metallic Cu.

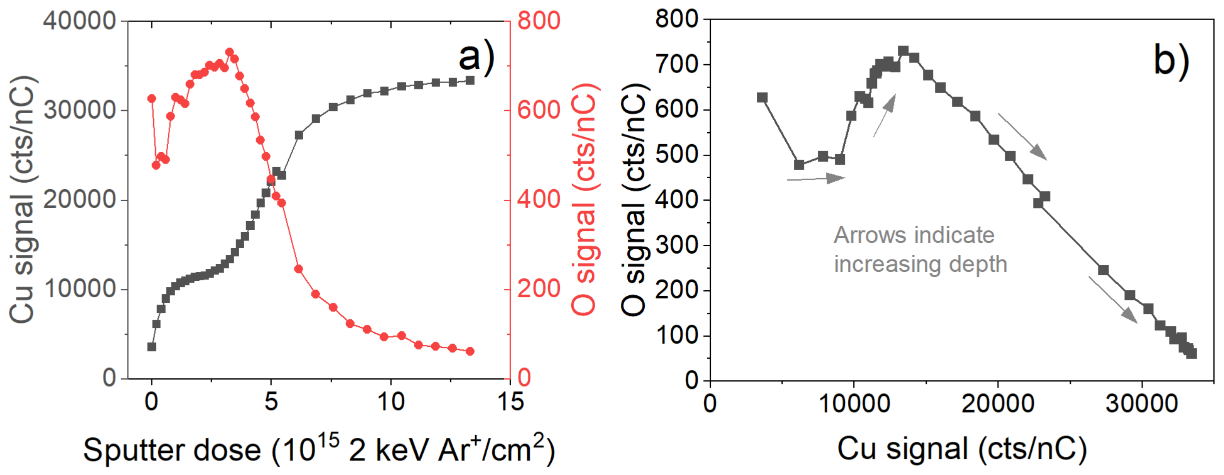

Figure S3. Sputter depth profile of a Cu foil taken from atmosphere and pretreated with 5 min atomic O. (a) 3 keV He^+ LEIS signal of Cu (left, black) and O (right, red) as a function of 2 keV Ar^+ sputter dose (He^+ spectra acquired alternating with Ar^+ sputter). (b) 3 keV He^+ signal from O (y-axis) plotted against Cu (x-axis), where each dot represents a progressive point of the depth profile in (a). In contrast to the Cu foil taken from atmosphere (Figure S2), there are nearly immediate Cu and O signals in the He^+ spectra (light recontamination from the vacuum is still initially removed, increasing total signal). After the initial Ar^+ dose, the relative composition of Cu and O is temporarily stable, suggesting a well-defined phase. The downturn in O signal is again correlated with breakthrough to pure Cu, which is approached asymptotically at high Ar^+ dose.

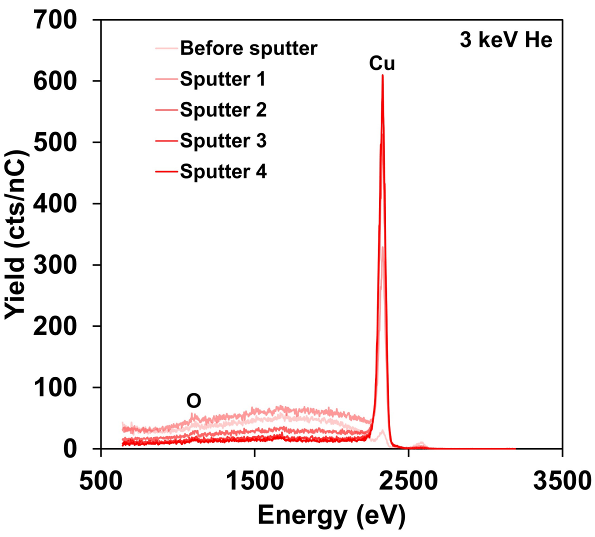

Figure S4. LEIS of pure Cu disk using 3 keV He^+ after consecutive 2 keV Ar^+ sputter cleaning cycles. Each cycle is 10 s with an estimated dose of 10^{15} Ar^+ ion/cm^2/cycle.

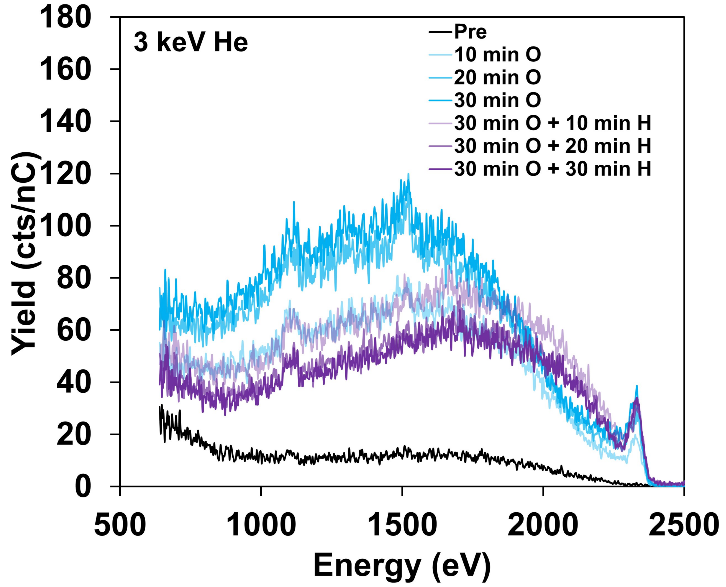

Figure S5. LEIS of pure Cu disk using 3 keV He^{+} as is (black), after 10 min, 20 min, and 30 min atomic O pretreatment (blue, decreasing transparency), and after 30 min atomic O, by 10 min, 20 min, and 30 min atomic H pretreatment (purple, decreasing transparency).

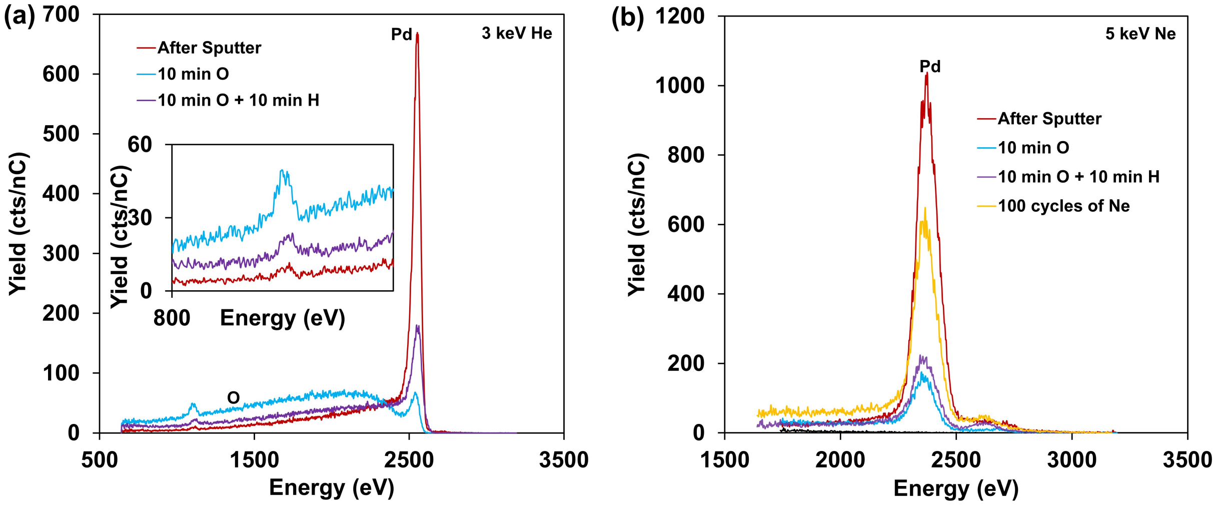

Figure S6. LEIS of pure Pd using (a) 3 keV He^{+} and (b) 5 keV Ne^{+}. Order of events between spectra: Sputter clean → 10 min at`omic O → 10 min atomic H → Ne^{+} depth profile (100 x 10^{13} ion/cm^{2}). Inset of (a) shows oxygen peak region more closely.

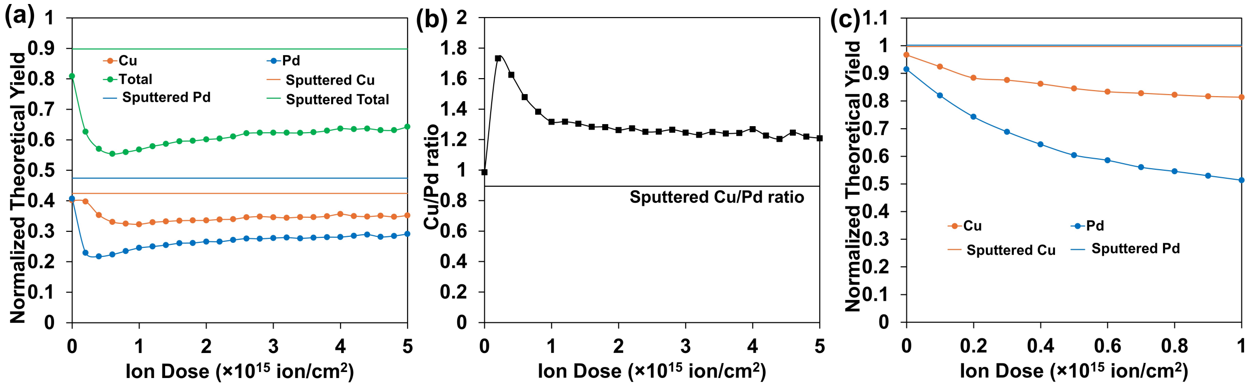

Figure S7. (a) 5 keV Ne^+ LEIS/profile of CuPd alloy after initial sputter cleaning by 2 keV Ar^+ (subsequent depth profile enacted intrinsically by repeated Ne^+ spectrum acquisition). Normalized theoretical yield (y-axis) refers to the signal intensity compared to the maximum signal determined by sensitivity factors of the sputter-cleaned pure metals. The horizontal solid lines refer to the Cu, Pd, Cu+Pd total normalized theoretical yield. While the sputtered alloy only reaches a total NTY of 0.9 based on the individual component sensitivity factors, this may in part be accounted by changes to facet distribution. (b) Cu/Pd ratio change during 500 cycles of Ne^+ depth profile. (c) 5 keV Ne^+ LEIS/profile of pure Cu and pure Pd after initial sputter cleaning by 2 keV Ar^+ showing the signal decay after sputter cleaning.

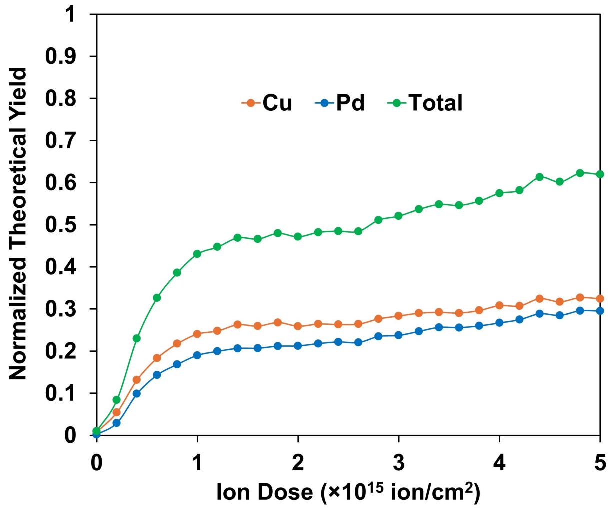

Figure S8. 5 keV Ne^+ depth profile of CuPd alloy introduced from atmosphere (with no pretreatment). Normalized theoretical yield (y-axis) refers to the signal intensity compared to the maximum signal determined by sensitivity factors of the sputter-cleaned pure metals.

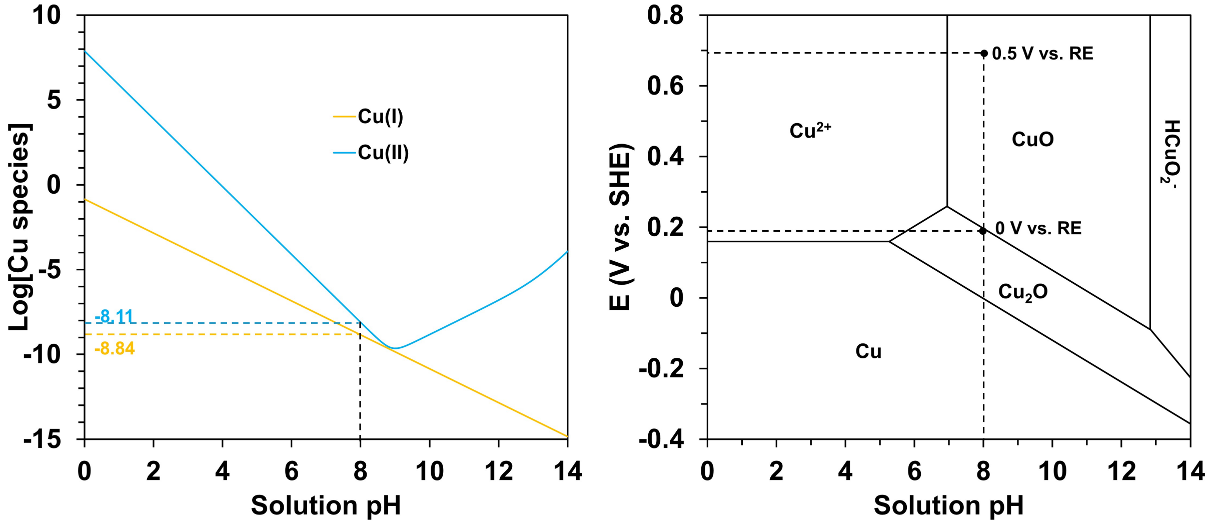

Figure S9. (a) Cu(I) solubility and Cu(II) solubility vs. pH; (b) Cu Pourbaix diagram. All thermodynamic data used to generate the plots taken from: Pourbaix, M. *Atlas of Electrochemical Equilibria in Aqueous Solutions*; National Association of Corrosion, 1974.

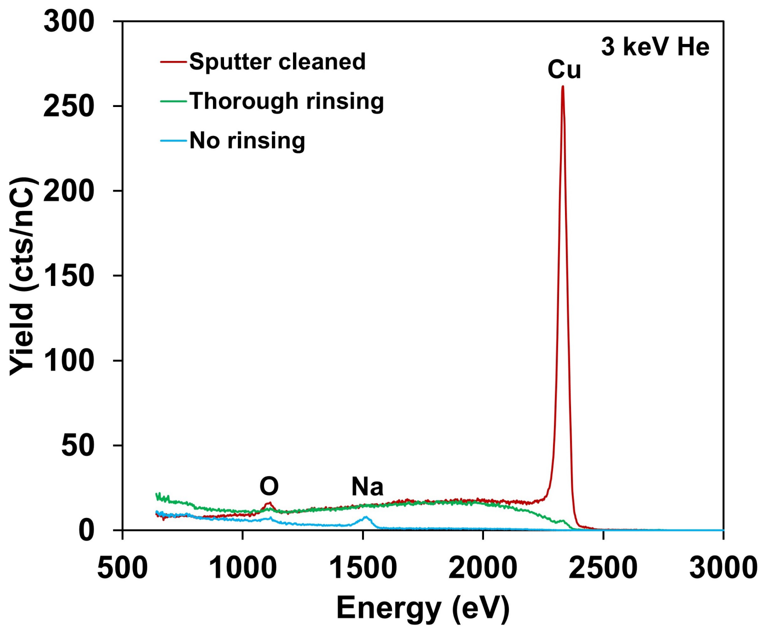

Figure S10. Comparison of 3 keV He^+ LEIS spectra of Cu disk after sputter cleaning (red trace), after immersing in 0.5 M $NaHCO_3$ solution for 10 min and transferring without rinsing (blue trace), and after immersing in 0.5 M $NaHCO_3$ solution for 10 min and thoroughly rinsing by 150 mL DI water before transferring to LEIS (green trace).

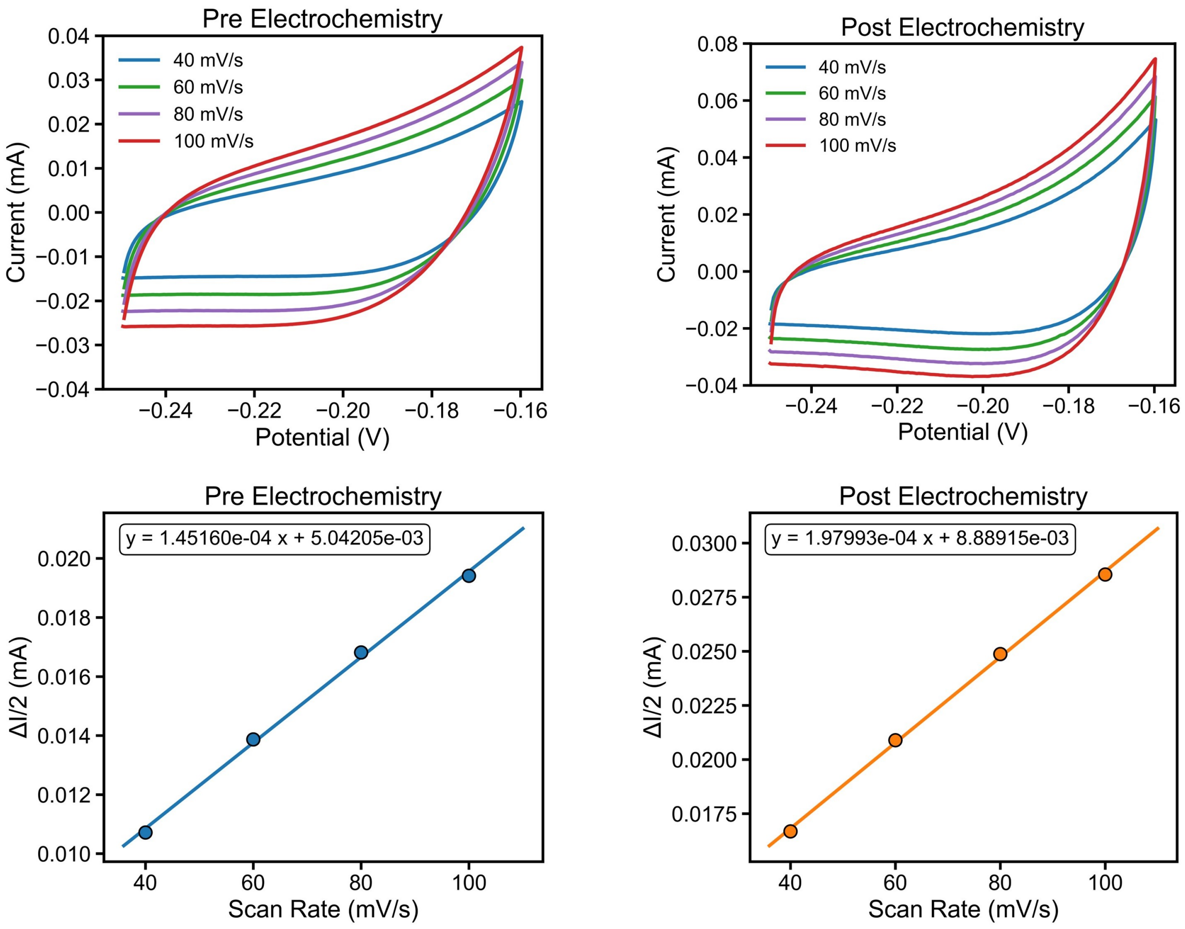

Figure S11. Electrochemical double-layer (ECDL) capacitance measurements of Cu disk electrode (a) before and (b) after cyclic voltammetry. The linear fits (c) before and (d) after CV are made from –0.22 V to –0.20 V, with slopes determining capacitance.

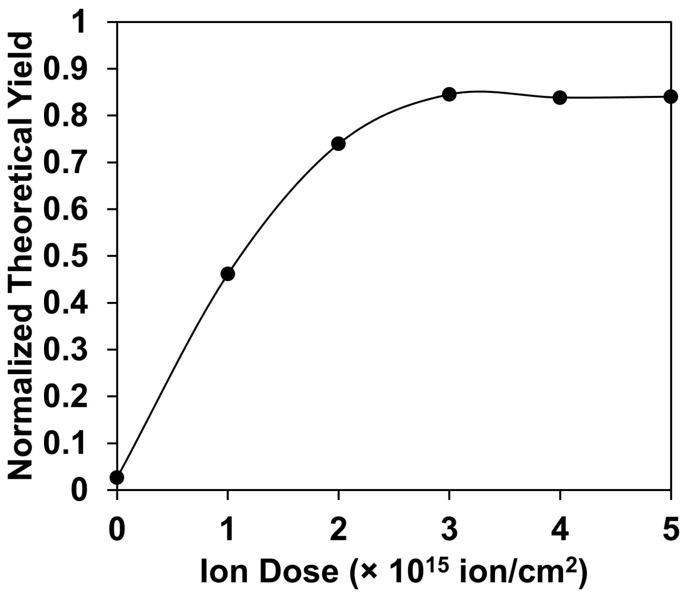

Figure S12. Cu quantification results from 5 keV Ne^+ LEIS milling cycle spectra (10^{13} ion/cm^2/cycle) of Cu electrode after -1.0 V hold for 10 min and transfer without potential control.

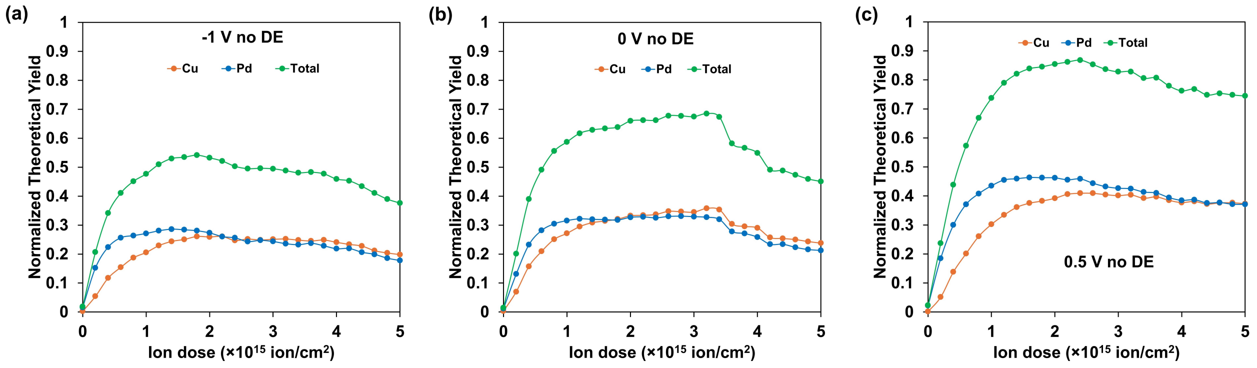

Figure S13. LEIS quantification of CuPd foil through 500 cycles of Ne^+ after (a) -1 V; (d) 0 V and (c) 0.5 V, all without the potential-controlled (dummy electrode) transfer. Normalized theoretical yield (y-axis) refers to the signal intensity compared to the maximum signal determined by sensitivity factors of the sputter-cleaned pure metals. Ne^+ dose per cycle: 10^{13} ion/cm^2.

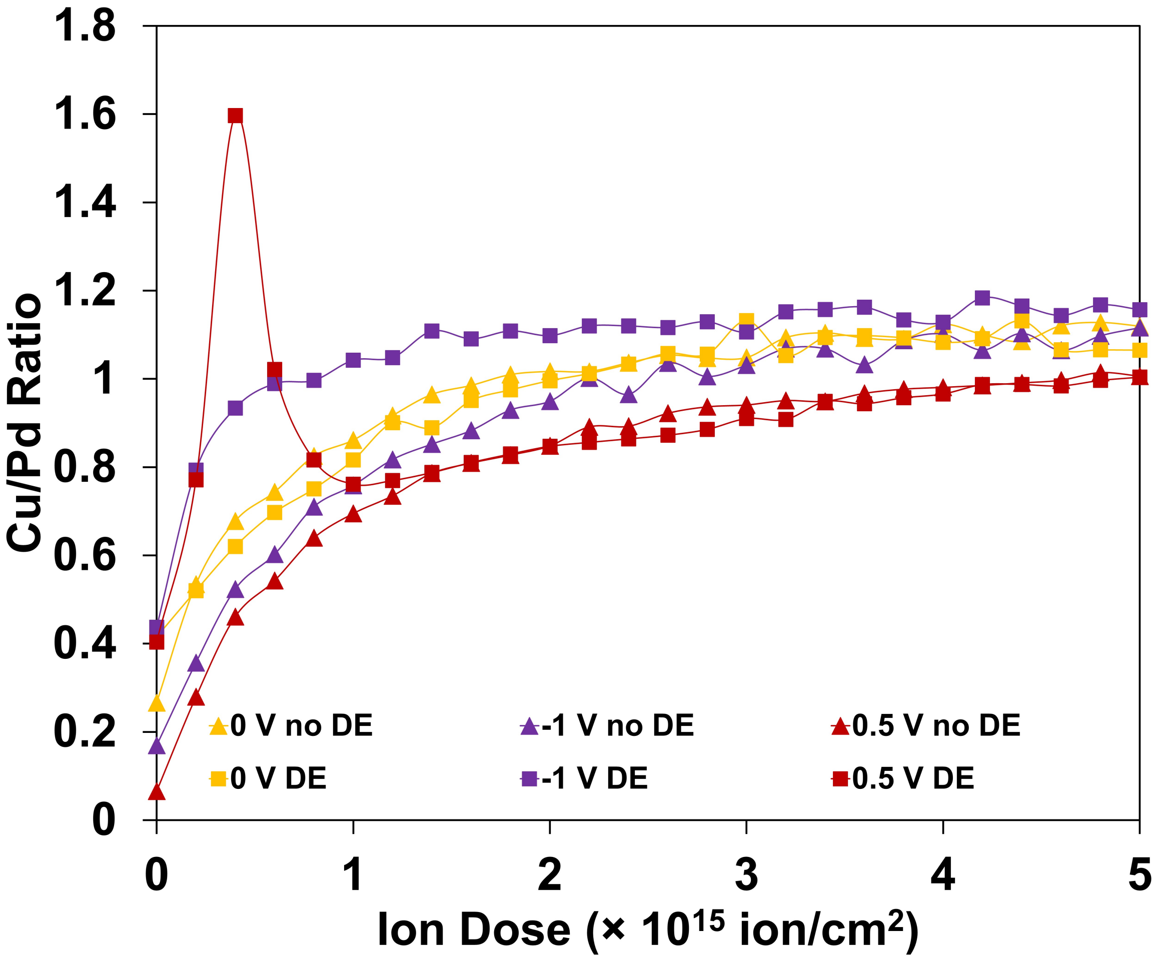

Figure S14. Comparison of Cu/Pd ratio with and without DE after electrochemical polarization on CuPd alloy surface in 0.5 M $NaHCO_3$ at different potentials (-1 V, 0 V, 0.5 V). Ne^+ dose per cycle: 10^{13} ion/cm^2. Square markers represent results with DE and triangular markers represent results without DE.
